# Due-B Is dispensable for early development and genome duplication in vertebrates

**DOI:** 10.1101/2025.06.17.658125

**Published:** 2025-06-17

**Authors:** Courtney G. Sansam, Emily A. Masser, Duane Goins, Christopher L. Sansam

**Affiliations:** 1. Oklahoma Medical Research Foundation, Cell Cycle and Cancer Biology Research Program, Oklahoma City, OK USA; 2. University of Oklahoma Health Sciences Center, Department of Cell Biology, Oklahoma City, OK USA

**Keywords:** development, Due-B, Replication, Sld2, zebrafish

## Abstract

The DNA Unwinding Element-Binding protein (DUE-B) is a Cyclin Dependent Kinase (CDK) and Dbf4-Dependent Kinase (DDK) substrate that has been implicated in the control of DNA replication initiation. Knockdown of DUE-B in HeLa cells perturbs the G1-to-S phase transition and immunodepletion of *due-b* in *Xenopus* egg extracts blocks replication initiation. Combined prior evidence has suggested that *Due-b* may be a vertebrate-specific DNA replication initiation factor. Here, we asked whether *due-b* was an essential vertebrate gene *in vivo*, and whether it was critical for proper embryonic development in the zebrafish *Danio rerio*. We have generated *due-b* mutant zebrafish through genome-editing TALENs that fail to express *due-b* mRNA or protein. Our mutant zebrafish are viable and survive to adulthood. They do not display outward developmental phenotypes, and when stressed with replication inhibitors, do not differ from their wild-type counterparts. Cell cycle analysis demonstrates that DNA replication occurs normally. Our data indicate that Due-b is not a core essential component of the DNA replication initiation machinery required for vertebrate development. Instead, it may be a mechanistically redundant protein or play a specialized role in DNA replication control.

## Summary

The DNA replication factor Due-b is not required for zebrafish development or genome duplication, suggesting it plays a redundant or specialized role in DNA replication.

## Introduction

DNA replication is strictly orchestrated so that the entire genome is replicated accurately and to completion during each cell cycle, prior to the onset of mitosis. Aberrant regulation of DNA replication is associated with primordial dwarfism, neuro-developmental disorders and genomic instability (1, 2). DNA replication is controlled at two steps: origin licensing and origin initiation. During origin licensing, the pre-replicative complex (pre-RC) comprised of CDT1, CDC6, and the MCM2-7 hexamer, forms at potential origin sites. Because more replication origins are licensed than will be used during S-phase, only a subset of origins are selected to fire and initiate the helicase. Therefore, the activation of the helicase requires the recruitment of additional replication factors to refine origin selection (3). In yeast, phosphorylation of the Mcm helicase by the Cdc7/Dbf4-dependent Cdc7 protein kinase (DDK) stimulates the recruitment of key initiation factors including Sld2, Sld3, and Sld7 to select licensed origins. S-phase specific phosphorylation of Sld2 and Sld3 by Cyclin Dependent Kinase (CDK) facilitate the association of additional replication proteins of the pre-initiation complex (pre-IC) at the selected origin. The Mcm helicase unwinds DNA, initiating DNA replication. The initiation proteins Sld2, Sld3, Sld7, and Dpb11 do not travel with the replication fork; however, these limiting replication proteins may be redistributed to other licensed origins to stimulate replication throughout the entirety of S-phase.

Vertebrates are thought to follow a similar mechanism for pre-RC and pre-IC formation, although the exact mechanisms remain unclear and there are notable differences in key replication protein orthologs. For instance, TRESLIN (also known as TICRR) is functionally analogous to Sld3, but it differs significantly in both structure and size, with only remote protein homology and no DNA sequence homology to the yeast Sld3 (4–6). MTBP, the metazoan Sld7, is triple the size of its yeast ortholog, although it still is an obligate binding partner to TRESLIN/Sld3. The functional ortholog of Sld2 remained the most elusive of the conserved core replication initiation factors. Once postulated to be RECQL4, more recent data indicates that DONSON fulfills the role of Sld2 in metazoans, as it is required for the assembly of the replicative helicase and interacts with both TRESLIN and TOPBP1 (7–9). In contrast to these poorly conserved initiation factors, other core replication proteins including CDC45, MCM2-7, and GINS, are highly conserved between yeast and vertebrates. Nevertheless, formation of a CMG complex CDC45-MCM-GINS, facilitated by TRESLIN/MTBP (Sld3/Sld7) and DONSON (Sld2), activate origin sites in both yeast and vertebrates (3, 7). Vertebrates have a more complex chromatin environment than yeast and larger genomes, so both additional proteins and additional protein-protein interactions likely play critical roles in the regulation of DNA replication (6, 10).

DNA unwinding elements (DUE) were identified in *E.coli* and in yeast as thermodynamically unstable sequences capable of helical unwinding (11). These unique sequence sites were proposed to serve as sites of replication initiation. The DNA Unwinding Element-Binding protein (DUE-B) was originally identified in a yeast one-hybrid screen for proteins affecting DNA replication initiation at the c-myc origin site (12). This well-characterized replication site contains a known zone of replication initiation and a DNA Unwinding Element that supports both transcription factor and replication factor binding (12–16). Deletion of the c-myc DUE region or deletion of DUE-B protein inhibited its origin activity (17). Chromatin-immunoprecipitation in HeLa cells confirmed the presence of DUE-B protein on chromatin at the c-Myc origin site, as well as at the Lamin-B2 core replicator origin site (12, 16).

Due-B is a 209 amino acid protein, identified as a non-essential D-aminoacyl-tRNA deacylase in *S. cereviase*. Intriguingly, vertebrate DUE-B has acquired an additional 60 amino acid C-terminal tail not evident in either *S. cerevisiae* or *C. elegans*. This C-terminal extension, hypothesized to complement activities of the yeast Sld3, interacts with TRESLIN, TOPBP1, and CDC45 proteins in HeLa cells (18, 19). Due-b binding to chromatin in *Xenopus laevis* extracts occurs prior to Cdc45 loading but independently of the pre-RC complex, as shown by experiments in which its depletion did not affect the proper loading of the pre-RC components (18, 19). Knockdown studies of DUE-B in HeLa cells delayed the G1-S transition and prevented both TRESLIN and CDC45 binding to chromatin (Poudel, 2018, Casper, 2004). Combined, these multiple lines of evidence underscore its putative role in the control of DNA replication initiation in vertebrates and suggest that DUE-B may be essential for proper development in vertebrates.

Here we report the generation and phenotypic analysis of a *due-b* mutant zebrafish. Given that Due-b has been reported to have essential functions in the loading of replication initiation proteins and the activation of replication origins in both mammalian cell culture and in *Xenopus* extracts, we asked to what extent it performed critical functions for DNA replication during embryonic development and whether *due-b* was an essential gene for vertebrate survival. The zebrafish is widely accepted as a particularly useful model system for studying vertebrate development. Zebrafish embryos develop outside the mother and are optically clear so that dividing cells can be readily observed. The external development of the embryos also allows for the direct introduction of targeted modifications through genome editing approaches (20–22). Zebrafish embryos contain stores of maternally deposited mRNA, allowing for the functions of core-essential proteins to be evaluated in early development prior to lethality. Zebrafish mutants of other replication proteins including, *ticrr* (*treslin*), *mcm3*, *gins*, and *rif1,* have been characterized for their roles in embryonic development and DNA replication in zebrafish (5, 23–26).

We successfully generated a TALEN-induced *due-b* mutant zebrafish carrying a frameshift mutation that results in severely reduced *due-b* mRNA levels and complete loss of detectable protein expression. Unexpectedly, these mutants are viable and survive to adulthood in Mendelian ratios. Maternal-zygotic *due-b* mutants, which lack both maternal and zygotic contributions, also survive and show no early developmental delays or defects. They exhibit no overt developmental phenotypes or cell division defects during embryogenesis, even when challenged with replication inhibitors. Cell cycle analysis and EdU incorporation confirm normal DNA replication, and early cleavage divisions proceed with normal timing. Although mutants display a modest reduction in eye size, they retain the ability to regenerate caudal fins, indicating that *due-b* is dispensable for DNA replication in both embryonic and adult proliferative contexts. Together, these results demonstrate that *due-b* is not an essential gene for genome duplication or development in zebrafish and suggest that it serves a redundant or specialized role in vertebrate replication control.

## Results

### TALEN induced frameshift mutation in due-B causes a sharp decreas in mRNA

**Figure 1:**
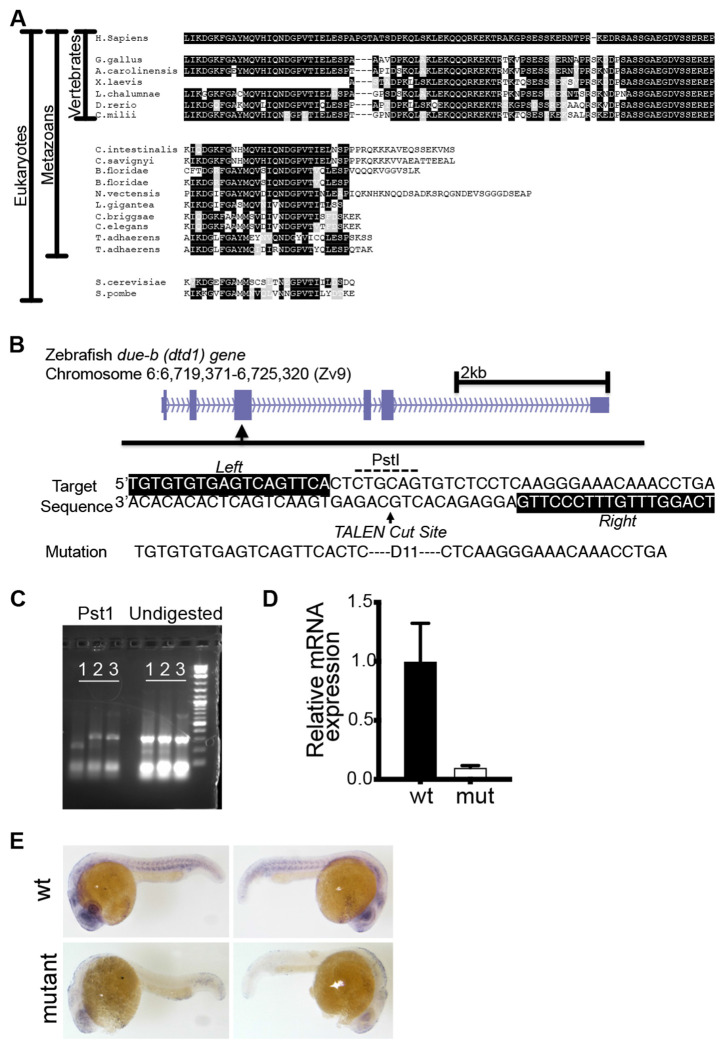
TALEN induced frameshift mutation in due-b causes a sharp decrease in mRNA. **(A)** Due-b C-terminal protein across species. The C-terminal extension is highly conserved but only found in vertebrates. **(B)** Schematic of TALEN designed to cut the *due-b* (also known as *dtd1*) gene on Chromosome 6 at an existing PstI restriction site. **(C)** We identified a Founder fish carrying an 11bp deletion. PCR amplification and digestion with PstI. Lanes show wt, het, and mutant in order. **(D)** Quantitative PCR shows sharp reduction in mRNA levels in mutant fish. **(E)** *In situ* hybridizations for due-b in wild-type and due-b mutant fish. due-b is expressed in the developing head, eye and central nervous system, while mutant fish display strong reduction in signal.

The C-terminal tail of human DUE-B is necessary for DNA replication in *Xenopus* egg extracts and interacts with TRESLIN, TOPBP1, and CDC45 (18). To assess the degree of conservation of the DUE-B C-terminal tail across species, we aligned the C-terminal protein sequences of Due-b from yeast to humans (Fig. 1A). Vertebrates, including zebrafish and frogs, exhibited a high degree of identity with the human DUE-B protein. Specifically, the zebrafish *Danio rerio due-b* C-terminal sequence shares 76% identity with the human version, while *Xenopus laevis* exhibits 78% identity.

Given the high conservation of the zebrafish Due-b sequence, we devised a strategy to generate a zebrafish mutant lacking the Due-b protein. The zebrafish has a single copy of the *due-b* gene (also known as *dtd1*) on Chromosome 6. Using TALENs (TAL-Effector-Like Nucleases) known for precise genome editing, we designed a targeted cut within exon 2 of *due-b*, focusing on a specific PstI restriction site (Fig 1B). We injected *due-b* site-specific TALENs into one-cell stage wild-type Tubingen AB5 (TAB5) zebrafish embryos, raised them to adulthood, mated them with wild-type TAB5 fish, and screened their F1 offspring for mutations. Through PCR amplification around the TALEN cut site and subsequent PstI digestion, we identified fish resistant to digestion, signifying a mutation at the restriction site sequence (Fig 1C, D). Sequencing confirmed that a zebrafish (F1-22) carried an 11bp deletion in *due-b*, leading to a frameshift mutation (Fig.1B). Typically, frameshift mutations in early exons trigger mRNA degradation via non-sense mediated decay, resulting in a deficiency of the encoded protein.

Confirmed F1-22 heterozygotes were bred to generate *due-b^+/+^*, *due-b*^+/*Δ11*^, and *due-b*^Δ11/Δ11^ clutch mates (hereafter referred to as *due-b^omf202/omf202^*). To assess *due-b* mRNA expression in these mutants, we genotyped individual embryos at 24 hours post fertilization (hpf), pooled like genotypes, and quantified mRNA levels using real-time PCR (qRT-PCR). Zebrafish embryos identified as *due-b^omf202/omf202^* exhibited a significant reduction in Due-b mRNA expression (Fig 1D). Next, we evaluated the extent of mRNA depletion throughout the whole embryo by performing *in situ* hybridizations of *due-b* in 24 hpf embryos. The *in-situ* results show that Due-b mRNA is highly expressed in the eye, brain, and along the developing central nervous system, all sites of rapid cell division at 24 hpf embryos (Figure 1E). The *due-b^omf202/omf202^* embryos lacked detectable mRNA expression at these sites (Figure 1E).

#### *due-b^omf202/omf202^* zygotic mutants are viable

**Figure 2:**
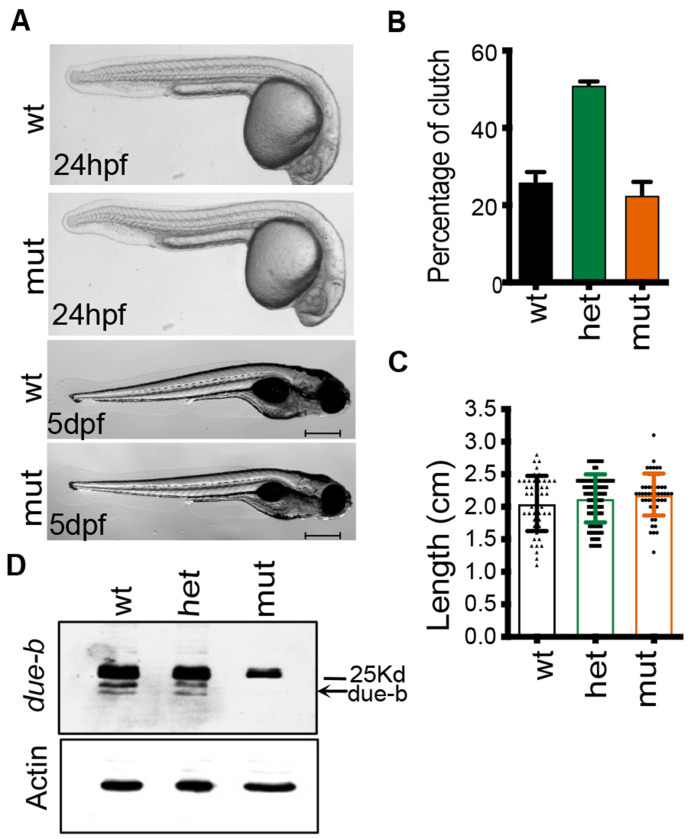
due-b^omf202/omf202^ zygotic mutants are viable. **(A)** Wild-type and due-b^omf202/omf202^ embryos at 24hpf and 5dfp do not show developmental differences. **(B)** Heterozygous due-b^+/omf202^ was incrossed to generate matched clutch mates. Normal Mendelian distributions were observed (n= 5 separate crosses). **(C)** Individual clutch mates from a Het incross were measured for length at Day 70 and data was evaluated using a One-Way ANOVA. **(D)** Western Blot for due-b protein in 10hpf embryos. B-actin loading control.

A heterozygous incross of zebrafish carrying mutations in components of the pre-RC such as *mcm3*, or of the pre-IC including *ticrr(treslin)* and *gins,* fail to survive past 5 dpf and show severe developmental deformities, including an underdeveloped head, severe spinal curvature, and small eyes (5, 24). From our original incross we observed that the *due-b^omf202/omf202^* mutant embryos survived through 24hpf, but we questioned whether they displayed any delays in development. Heterozygote F1-22 zebrafish were mated and the progeny raised to evaluate long-term survival and developmental growth. We carefully monitored *due-b^omf202/omf202^* clutches for survival and developmental deformities throughout the first few days of development (Fig. 2A). Unexpectedly, *due-b^omf202/omf202^* embryos were viable, showed no overt developmental phenotype at 24hpf, and were free-feeding by 5dfp. The *due-b^omf202/omf202^* zebrafish reached sexual maturity at expected Mendelian ratios (Fig 2B). By Day 70, we measured the length of each fish to determine if *due-b^omf202/omf202^* zebrafish matured with an overall smaller size. While wild-type and heterozygous fish had mean lengths of 2.05cm (*n=47*) and 2.13cm *(n=100*, respectively, *due-b^omf202/omf202^* zebrafish were similarly sized, with a mean length of 2.19cm *(n=52)*. The differences in overall length did not reach statistical significance based on genotype (One-way ANOVA, Fig 2C). To confirm whether *due-b* protein was absent in these mutants, we collected synchronized wild-type, heterozygous, or *due-b^omf202/omf202^* embryos at 10hpf. We dechorionated and deyolked the embryos, disaggregated the cells, and made whole cell protein lysate using RIPA buffer and immunoblotted for Due-b (antibody gift of M. Leffack). While a band of 24 kDa was detected readily in the wild-type sample, the *due-b^omf202/omf202^* embryos lacked this band, confirming loss of *due-b* protein product (Figure 2D).

### Maternal loss of Due-b does not affect development

**Figure 3:**
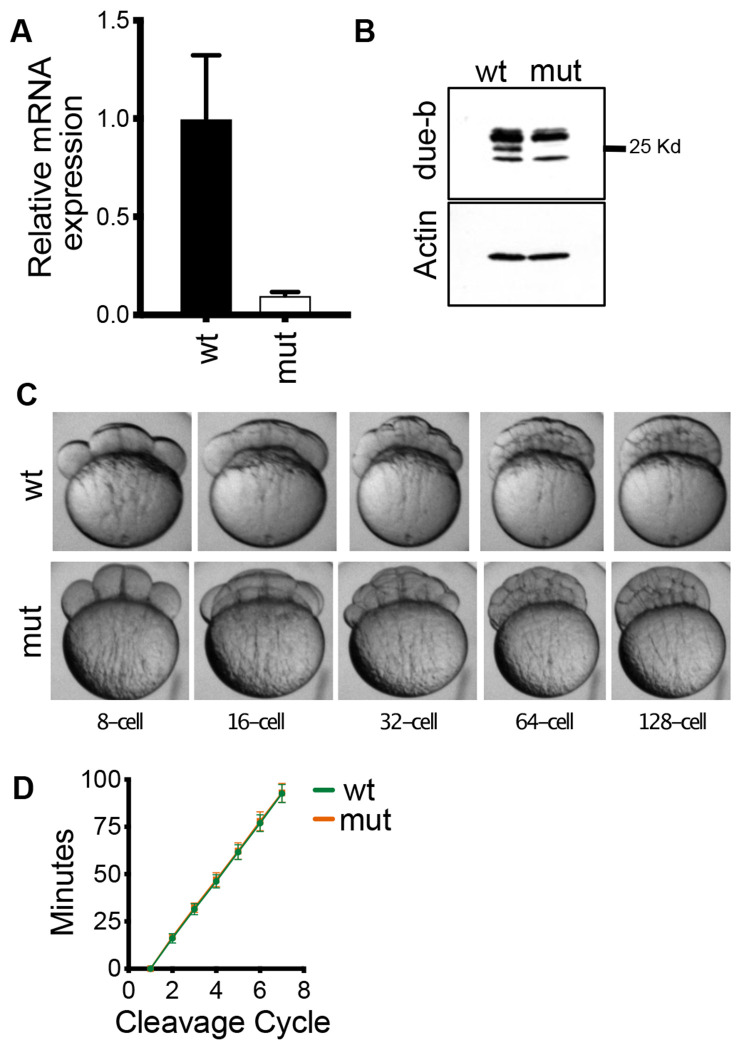
Maternal loss of due-b does not affect early development. **(A)** Quantification of total mRNA expression shows that the levels of due-b mRNA are greatly reduced in *due-b* maternal zygotic mutants. **(B)** Western Blot for due-b protein in 10hpf wild type or maternal zygotic mutant embryos. B-actin loading control. (**C, D)** Synchronized wild-type or mutant embryos were imaged for the first eight cell divisions and the timing of cleavage events was recorded. No differences in cell division time was observed (n=10 wt embryos, n=11 mutant embryos).

We were concerned that the lack of overt phenotype observed in the *due-b^omf202/omf202^* embryos could be a consequence of maternal mRNA contributions supplied by the mother in the first stages of development. Unlike mammals, zebrafish and frog embryos develop outside the mother and do not commence zygotic transcription until the mid-blastula transition (MBT) at approximately the 1000 cell stage (3hpf). Thus, embryos can survive off maternally deposited mRNAs in the yolk for up to 5 days post fertilization (dpf), until maternal mRNA stores are completely depleted. To test whether maternal *due-b* mRNA masks early developmental defects in the *due-b^omf202/omf202^* embryos, we mated an adult homozygous *due-b^omf202/omf202^* zebrafish to generate maternal-zygotic mutants, which lack maternal mRNA contribution. To confirm efficient depletion of *due-b* transcripts in these embryos, we collected synchronized wild-type TAB5 and *due-b^omf202/omf202^* embryos at 6 hpf (shield stage) and performed quantitative RT-PCR. As observed in the zygotic mutants, *due-b* mRNA levels were nearly undetectable in maternal-zygotic embryos compared to wild-type controls (1 ± SD 0.117 vs. 0.04 ± SD 0.0084) (Fig. 3A). We also assessed protein expression at 10 hpf and again found that the 24 kDa Due-b protein was readily detected in wild-type lysates but absent in the mutant embryos (Fig. 3B).

Zebrafish embryos, from fertilization at 0hpf to 3hpf, are remarkable for their synchronous, rapid cleavage cycles. These early cleavage events are continuous cycles of DNA replication followed by mitosis without gap phases (G1 and G2). Cleavages occur every 15 minutes until cell cycle lengthens following the 10^th^ cell division at the MBT. The lengthening of S phase at this timepoint is accompanied by a loss of synchrony in cell divisions (27). We reasoned *due-b* may be critical for these early rounds of DNA replication, as it has been reported that loss of functional *due-b* impairs loading of Cdc45 onto chromatin in *Xenopus* extracts and in HeLa cells. It was possible that the early cell cycles were compromised, even though the embryos ultimately recovered and survived to adulthood at expected ratios. Detecting such defects are a particular strength of the zebrafish system. We therefore monitored these early cleavage cycles in wild type and *due-b^omf202/omf202^* maternal-zygotic mutant embryos by time-lapse photography (Fig. 3C). We timed the cell cleavage events in each embryo and plotted these division cycles against time (Fig. 3D). Our results show identical cleavage cycle times for wild-type and *due-b* mutant embryos indicating that loss of the *due-b* protein does not impair these early developmental cycles.

### Normal DNA replication in *due-b* mutant embryos

**Figure 4:**
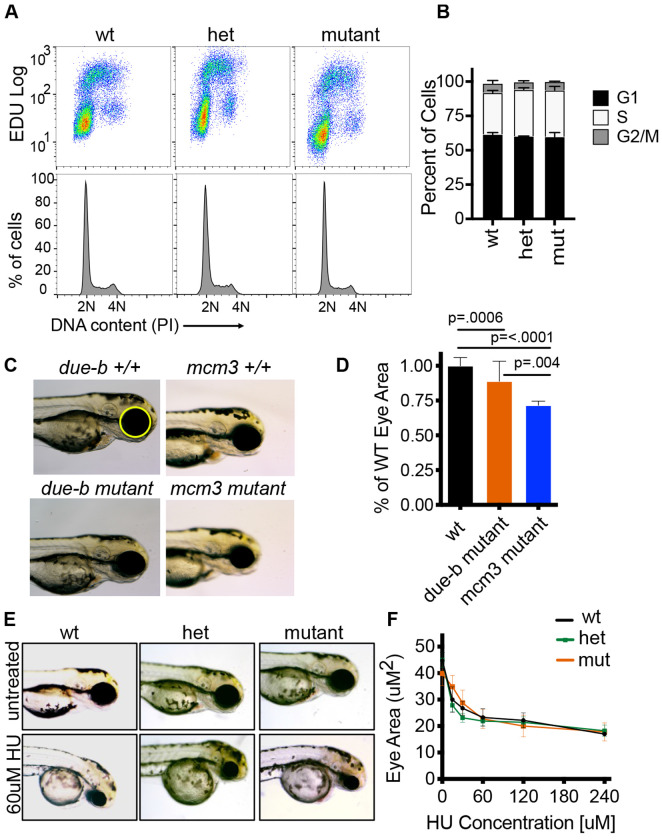
Marginal DNA replication defects in due-b mutants. **(A)** EdU incorporation and propidium iodide (PI) flow cytometry analysis of cells from pools of EdU pulse-labeled 24hpf wild-type, heterozygous, and *due-b* mutant embryos (n=20,000 cells evaluated). **(B)** Quantification of flow cytometry data. Cells with G1, S, or G2/M DNA content do not show any difference in cell cycle phases between genotypes. **(C**) Eye area images of 72hpf wt and *due-b* mutant embryos (left) or wt and *mcm3* mutant embryos (right). **(D)** Quantification of eye area was performed using ImageJ. Eye area was normalized to wild-type fish eyes. Statistical analysis using One-Way ANOVA showed significant differences between wt and *mcm3* mutants (p<0.0001), between wt and *due-b* mutant (p=.0006) as well as between *mcm3* mutants and *due-b* mutants (p=.0041). **(E,F)** 24hpf embryos were placed in E3 fish water containing different concentrations of hydroxyurea, ranging from 0uM to 240uM. The eye area was imaged and quantified at 72hpf. There was no difference in eye area between genotypes in response to hydroxyurea treatment.

We next wanted to directly assess DNA replication in the *due-b* mutants. To do this, we evaluated their ability to replicate DNA using the thymidine analog 5-ethynyl-2’-deoxyuridine (EdU). At 24 hpf, wild-type, heterozygous, and *due-b* mutants were incubated in 10mM EdU. After a 15-minute recovery period in E3 fish water, the embryos were disaggregated and prepared for flow-cytometry using click-chemistry to quantify the EdU and propidium iodide to detect the DNA content (Fig 4A). Surprisingly, embryos deficient for *due-b* showed no impairment in the ability to incorporate EdU. The distributions of cells into different phases of the cell cycle were consistent across genotypes, indicating normal DNA replication (Fig 4B).

### Developmental eye growth is largely preserved in *due-b* mutants

The developing zebrafish eye is one of the most prominent and rapidly growing organs during early embryogenesis, with extensive proliferation and tissue expansion occurring between 16 and 72 hpf (24). Because this growth depends on robust DNA replication, measurements of eye size can serve as a sensitive readout of proliferative capacity during development. We therefore asked whether *due-b* mutant embryos exhibit reduced eye size as an indicator of compromised replication. To address this, we imaged and measured the eye area of individual 72 hpf *due-b^omf202/omf202^* embryos and compared them to wild-type siblings (Fig. 4C–D). As a benchmark for replication-dependent eye growth, we also analyzed 72 hpf embryos from an *mcm3*^^+/*HI3068*^ incross. MCM3 is a core component of the replicative helicase within the pre-RC, and loss of *mcm3* has been shown to impair eye development (24). Quantification revealed that *due-b* mutants had a modest but statistically significant reduction in eye area (49.1 μm, *n* = 45) compared to wild-type embryos (53.4 μm, *n* = 28; *p* = 0.0004, one-way ANOVA). By comparison, *mcm3* mutant embryos exhibited a more pronounced phenotype, with an average eye area of 43.7 μm (*n* = 7) (Fig 4D).

### Due-b loss does not increase hydroxyurea sensitivity

Having shown little or no effect on proliferation during development in *due-b* mutant embryos, we next asked whether replication stress might uncover a more subtle role for *due-b* in DNA replication. Only a fraction of licensed replication origins are normally activated during S phase, while the remainder function as dormant origins that can be used when replication forks stall. Hydroxyurea (HU) induces replication stress by inhibiting ribonucleotide reductase, leading to dNTP depletion and fork stalling, thereby increasing reliance on dormant origin activation. Sensitivity to HU is a hallmark of mutations that impair replication initiation or fork stability. To test whether *due-b* is required under these conditions, we treated wild-type and *due-b^omf202/omf202^* embryos with increasing concentrations of HU from 24 to 72 hpf and measured eye size as a readout of cellular proliferation during DNA replication stress. HU reduced eye size in both genotypes in a dose-dependent manner, but *due-b* mutants did not show increased sensitivity, suggesting that dormant origin usage remains largely intact in the absence of *due-b* (n = 8 per genotype; Fig. 4E–F).

### Adult zebrafish are able to regenerate caudal fin in absence of Due-b.

**Figure 5:**
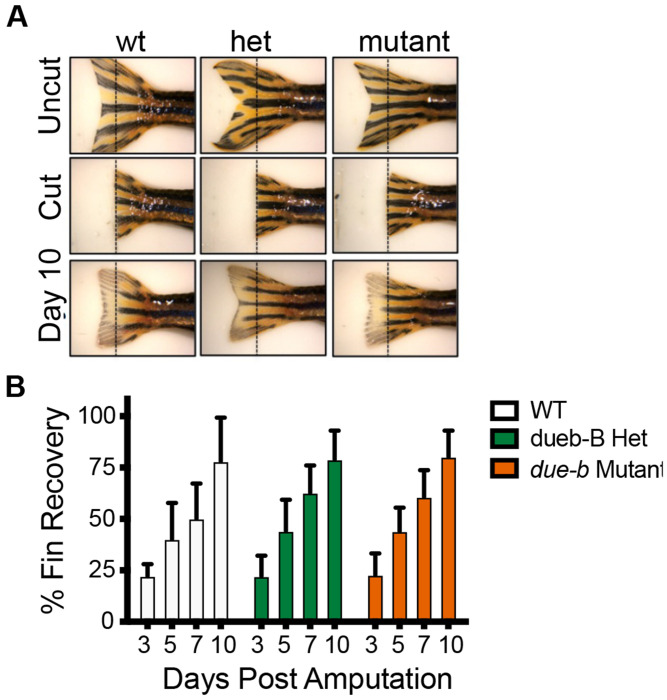
Adult zebrafish are able to regenerate caudal fin in absence of due-b. **(A)** The caudal fins of adult zebrafish were photographed, cut, and photographed again at day 0 (post -clip) 3, 7, and 10 days. Fin regrowth was measured as the number of pixels/mm2 of a line from the cut position to edge of new growth using image J. **(B)** The percent of re-growth was calculated from the difference for wt (n=7), heterozygous (n= 18) and mutant fish (n=12).

Until this point, we had focused on DNA replication in the development of the early embryo. We next considered the possibility that *due-b* may be essential for DNA replication in proliferating cells of adult tissues. We reasoned that a critical role of *due-b* could be in the re-initiation of DNA replication from quiescent, non-cycling cells. The zebrafish has the remarkable capability of regenerating many tissues, and regeneration of an amputated caudal tail fin in zebrafish is well characterized and easily monitored (28–30). Importantly, the zebrafish tail fin is comprised of terminally differentiated non-muscularized cells in the adult fish. To regenerate the tail following amputation, cells adjacent to the amputation site de-differentiate and cells of the blastema must re-enter the cell cycle and initiate rounds of DNA replication. Therefore, we asked if *due-b* played a role in regenerating tissue of the caudal fin. We amputated the caudal tail fins of wild-type, heterozygous, and *due-b* mutant (n=40), photographing each fish before and after surgery. We then single-housed the fish in tanks held at 33°C for 10 days. We photographed each fish on days 3, 5, 7, and 10 post-surgery and quantified the percent of tail regrowth (Fig 5A–B). All the fish were able to regenerate their tail fins, and we found no significant difference in the rate of tail regeneration between wild-type and *due-b* mutant fish. This data indicates that Due-b is not required for DNA replication re-initiation from non-cycling cell populations.

## Discussion

We chose to target the *due-b* gene for mutagenesis in zebrafish because of its proposed role in DNA replication initiation. Studies in *Xenopus* egg extracts and human cell culture showed that DUE-B loss delays the G1–S transition and disrupts CDC45 loading onto chromatin (12, 18, 19, 31, 32). The unique C-terminal extension of DUE-B, which is absent in yeast, is essential for replication function in human cells and in *Xenopus* egg extracts (31, 32). DUE-B also interacts with the MCM2-7 complex, CDC45, TRESLIN, and TOPBP1, all of which are well-established replication initiation factors (18, 19).

Because zebrafish mutants of replication initiation genes display severe phenotypes, we expected that *due-b* loss would cause similar developmental defects. Instead, our results show that *due-b* loss does not overtly impact viability or S-phase progression in zebrafish. Mutant embryos survive to adulthood at normal body size. This contrasts with replication protein mutants like *mcm3*^HI3068^ and *ticrr*^HI1573^, which are embryonic lethal and show a range of pleiotropic developmental phenotypes, including small head and eyes, body curvature, and apoptosis (5, 24). Although the *due-b* mutants have a modest reduction in eye size, the effect is much less severe than that observed in *mcm3* or *ticrr* mutants, suggesting a minimal or no impact on DNA replication. Despite the dose-dependent sensitivity of eye development to hydroxyurea, *due-b* mutants did not exhibit a more pronounced eye defect following treatment. Finally, we confirmed that *due-b* mutants have normal adult caudal fin regeneration, a process that depends on efficient DNA replication during the proliferation of blastema cells immediately after amputation (33). This further supports the conclusion that zebrafish *due-b* is unnecessary for genome duplication in embryos or adults.

Although we do not know why our results differ from published findings in *Xenopus* and HeLa cells, the discrepancy may reflect differences in developmental timing, cellular environment, or compensatory mechanisms specific to zebrafish. One possibility is that the DNA replication function of Due-B emerged after the divergence of teleosts and tetrapods, but this seems unlikely, as the C-terminal domain, which is absent in yeast and is essential for replication in other systems, is conserved in zebrafish. Moreover, genome-wide CRISPR screens in human cell lines have also failed to identify DUE-B as universally necessary for cellular proliferation or fitness, indicating that its role also may be cell-type specific or conditionally required in humans (34, 35). It is possible that DUE-B becomes more crucial in pathological settings such as cancer. For instance, DUE-B could be required under oncogene-induced replication stress, when origin licensing and firing are dysregulated (36). It may serve to stimulate non-preferred origin sites or buffer against loss of other replication factors. These context-specific roles could explain why DUE-B is critical in certain HeLa cell assays but dispensable in the zebrafish embryo.

Another possibility is that genetic compensation masks the effects of *due-b* loss. This phenomenon, documented in zebrafish, can obscure phenotypes seen with knockdowns (33). Compensation may occur through transcriptional or pathway-level responses to gene disruption. Although we did not directly test for upregulation of other factors involved in replication initiation, such as TRESLIN, MTBP, DONSON or RECQL4. Genetic compensation for DUE-B mutation may also occur in humans, as genome-wide CRISPR screens in human cells also show that DUE-B knockout has minimal fitness effects compared to other core replication factors (34, 35). Additional work will be needed to determine whether DUE-B’s role in replication is highly context dependent, or whether knockouts allow sufficient time for compensatory responses not seen in acute knockdown or inhibition studies.

The evolution of additional replication factors in metazoans likely reflects the demands of replicating large genomes embedded in complex chromatin. Regulatory proteins help coordinate replication timing and domain structure (27, 37). It is possible that DUE-B functions in a more specialized manner, acting at specific origins or in distinct chromatin contexts. In that case, the variable essentiality of DUE-B might reflect shifts in replication origin usage rather than rewiring of the core initiation process itself. This remains speculative, but there is emerging evidence that regulators such as RECQL4 can differentially control efficient versus dormant origins (38). Although we cannot yet define when or where DUE-B is required for origin firing, our results show clearly that it is not essential for DNA replication during key stages of zebrafish development.

## Materials and methods

### Animal care

Zebrafish were housed and cared for in strict accordance with protocols approved by the OMRF Institutional Animal Care and Use Committee. Adult fish were housed in an aquatic facility in tanks at a density of 10 fish per liter and maintained at a constant 26.5°C with 10-hour light and 14-hour dark cycles.

### TALEN production and microinjection

The TALEN expression constructs targeting the third exon of the *due-b (dtd1)* gene were assembled in pCS2TAL3-DD and pCS2TAL3-RR using Golden Gate Assembly as in (39). pCS2TAL3-DD and pCS2TAL3-RR were gifts from David Grunwald (Addgene plasmids # 37275 and 37276; RRID:Addgene_37275; RRID:Addgene_37276). mRNA encoding the TALE nuclease subunits was synthesized using the mMESSAGE mMACHINESP6 Transcription Kit (AM1340; ThermoFisher Scientific) and purified using RNeasy Mini Kit (74104; Qiagen). 125pg of each TALEN mRNA was injected into 1-cell stage zebrafish embryos. TALEN-injected embryos were raised to adulthood and assessed for transmission of insertions/deletions at the TALEN cut site by Pst1 restriction digest of PCR amplicons. F1 fish from mutation transmitting F0 animals were raised to adulthood and analyzed for mutation by PCR (*Forward primer*: tgtaatacgactcactatagggCAGGTTTTTCATCCCTGCAT; Reverse Primer: aaaacgacggccagtCTGCTCCAGCATGTTGTTGT) and restriction digest. The specific *due-b* mutation was identified by Sanger sequencing.

### Genotyping

Wild-type, heterozygous, and *due-b^omf202/omf202^* genotypes were identified by PCR analysis using the following primers: CS929: CTGGAGCAGCTTAGAGAAACCT; CS930: TGTGTGAGTCAGTTCACTCTG; CS931: GTGTGAGTCAGTTCACTCCT; CS791: GCGTTTAACAACAGTAGGCAATCA. All primers were purchased from Integrated DNA Technologies.

### *in situ* hybridization

*In situ* hybridization protocol was followed as in (40). Briefly, ISH probes were constructed by PCR from genomic DNA and isothermal assembly into HindIII-BamHI digested pUC57-Amp plasmids flanked byT3 andT7 promoters for sense (coding RNA) and anti-sense strand synthesis, respectively. Primers used to amplify probe sequences are as follows: *due-b* forward: accctcactaaagggaaGAGCGAGCGTAACAGTTGGA;

*due-b* reverse: acgactcactatagggcTGGCGCTGGGATCTACTTTT. Dioxygenin (DIG)-labelled RNA probes were synthesized with T3 and T7 polymerase with reagents from Roche. Embryos were manually dechorionated with forceps, then fixed in 4% paraformaldehyde in 1X PBS overnight. Fixed embryos were dehydrated in 100% methanol and stored at −20°C. Embryos were proteinase K treated for 10 minutes. The protocol was programmed into an Intavis VSi machine to automate the prehybridization and washing procedures. Embryos were removed from the machine and incubated in DIG-labelled RNA probes overnight in a 70°C water bath. Embryos were then returned to the machine for washing, pre-incubation with Bovine Serum Albumin, incubation with anti-DIG antibody, and additional washing. Labelled embryos were imaged with a Nikon SMZ1500 stereomicroscope with Andor Zyla CMOS camera.

### Quantitative RT-PCR

RNA was isolated from 50-80 embryos using Tri-Reagent (Invitrogen AM9738) following protocol from (41). mRNA was converted to cDNA using the AccuScript High Fidelity Reverse Transcriptase (600089; Agilent, Santa Clara, CA). qPCR was done on a Roche LightCycler 480 with the SYBR Green I Master (04707516001; Roche) master mix. Three technical replicates were performed for each sample. The relative mRNA expression was calculated using the 2-ΔΔ^Cq^ method. The following primer sequences were used: *due-b* forward: CGGGCAGTTTGGAGCAAAAA; *due-b* reverse: TGGACAGCAGTTTGGGATCT; r*pl13a*: TCTGGAGGACTGTAAGAGGTATGC; *Rpl13a* reverse AGACGCACAATCTTGAGAGCAG.

### Western blot

Bud stage embryos (10hpf) were collected, dechorionated with pronase (Roche 11459643001) deyolked, and lysed in RIPA buffer (150mM NaCl, 50mM Tris pH8, 1mM EDTA, 0.5% deoxycholate, 1%NP-40, 0.1%SDS). 50ug of total protein were loaded onto SDS-PAGE gel. Anti-DUE-B antibody was a gift of Michael Leffack. The B-Actin Antibody was purchased from Abcam (ab6276).

### Cell cycle analysis

EdU experiments were based on Sansam 2010 (5). Briefly, after fertilization, individual embryos were placed in single wells of 96-well plate. At 24hpf, embryos were dechorionated with pronase (Roche 11459643001), gently washed in E3 medium (5mM NaCl, 0.17mM KCl, 0.33mM CaCl2, 0.33mM MgSO4). They were labelled with 20mM 5-ethynyl-2’-deoxyuridine (EdUThermoFisher A10044) in DMSO for 20 minutes, transferred to fresh E3 medium and allowed incubated for an additional 15 min at 28.5°C. Embryos were then washed thoroughly in 1XPBS and placed in ice. Embryos were disaggregated with a multi-channel pipet into a single cell suspension and a small portion of cells were removed for genotyping PCR. The remaining cells were fixed in 70% ethanol overnight. Cell suspensions from the same genotype were pooled and prepared for flow-cytometry with the click-chemistry. Flow cytometry data was quantified by FlowJo (TreeStar, Inc.)

### Tail fin regeneration

Tail fin regeneration assays were performed as in described in (30).

## Data Availability

Flow cytometry data will be made available in a public repository upon publication.
